# Effect of NK-5962 on Gene Expression Profiling of Retina in a Rat Model of Retinitis Pigmentosa

**DOI:** 10.3390/ijms222413276

**Published:** 2021-12-10

**Authors:** Shihui Liu, Mary Miyaji, Osamu Hosoya, Toshihiko Matsuo

**Affiliations:** 1 Department of Ophthalmology, Graduate School of Interdisciplinary Science and Engineering in Health Systems, Okayama University, Okayama City 700-8558, Japan; shihuiliu@okayama-u.ac.jp; 2 Department of Medical Neurobiology, Graduate School of Medicine, Dentistry and Pharmaceutical Sciences, Okayama University, Okayama City 700-8558, Japan; mmiyaji@okayama-u.ac.jp (M.M.); hosoya@okayama-u.ac.jp (O.H.)

**Keywords:** apoptosis, drug, retina, photoreceptors, retinitis pigmentosa, extracellular exosome, extracellular matrix organization, *PI3K–Akt* signaling pathway, *SERPINF1*, pigment epithelium-derived factor (PEDF)

## Abstract

Purpose: NK-5962 is a key component of photoelectric dye-coupled polyethylene film, designated Okayama University type-retinal prosthesis (OUReP™). Previously, we found that NK-5962 solution could reduce the number of apoptotic photoreceptors in the eyes of the Royal College of Surgeons (RCS) rats by intravitreal injection under a 12 h light/dark cycle. This study aimed to explore possible molecular mechanisms underlying the anti-apoptotic effect of NK-5962 in the retina of RCS rats. Methods: RCS rats received intravitreal injections of NK-5962 solution in the left eye at the age of 3 and 4 weeks, before the age of 5 weeks when the speed in the apoptotic degeneration of photoreceptors reaches its peak. The vehicle-treated right eyes served as controls. All rats were housed under a 12 h light/dark cycle, and the retinas were dissected out at the age of 5 weeks for RNA sequence (RNA-seq) analysis. For the functional annotation of differentially expressed genes (DEGs), the Metascape and DAVID databases were used. Results: In total, 55 up-regulated DEGs, and one down-regulated gene (*LYVE1*) were found to be common among samples treated with NK-5962. These DEGs were analyzed using Gene Ontology (GO) term enrichment, Kyoto Encyclopedia of Genes and Genomes (KEGG), and Reactome pathway analyses. We focused on the up-regulated DEGs that were enriched in extracellular matrix organization, extracellular exosome, and *PI3K–Akt* signaling pathways. These terms and pathways may relate to mechanisms to protect photoreceptor cells. Moreover, our analyses suggest that *SERPINF1*, which encodes pigment epithelium-derived factor (PEDF), is one of the key regulatory genes involved in the anti-apoptotic effect of NK-5962 in RCS rat retinas. Conclusions: Our findings suggest that photoelectric dye NK-5962 may delay apoptotic death of photoreceptor cells in RCS rats by up-regulating genes related to extracellular matrix organization, extracellular exosome, and *PI3K–Akt* signaling pathways. Overall, our RNA-seq and bioinformatics analyses provide insights in the transcriptome responses in the dystrophic RCS rat retinas that were induced by NK-5962 intravitreal injection and offer potential target genes for developing new therapeutic strategies for patients with retinitis pigmentosa.

## 1. Introduction

Retinitis pigmentosa (RP) is a hereditary disease that causes blindness due to the loss of retinal photoreceptor cells. Patients with RP experience slowly progressive loss in the peripheral visual field, finally leading to blindness in later decades [1]. Nowadays, many treatments including neurotrophic factors [2,3], antioxidants [4,5,6], retinal prostheses [7,8,9,10,11,12], and gene therapies [13] are used to rescue retinal degeneration and improve the visual function.

RCS rats were used as an animal model of RP in many previous studies. In the RCS rat, a 409 bp deletion in the receptor tyrosine kinase *MERTK* gene mutation leads to reduced phagocytic function of the retinal pigment epithelial (RPE) cells and causes accumulation of photoreceptor outer segment debris in the subretinal space. Later, this debris blocks efficient oxygen and nutrient transport to photoreceptor cells and then leads to progressive photoreceptor degeneration and subsequent vison decline [14,15,16]. Photoreceptor cells in the RCS rats begin to degenerate on postnatal day (P) 22. Apoptosis of photoreceptors reaches its peak on P32, and then it gradually decreases [17].

**Figure 1:**
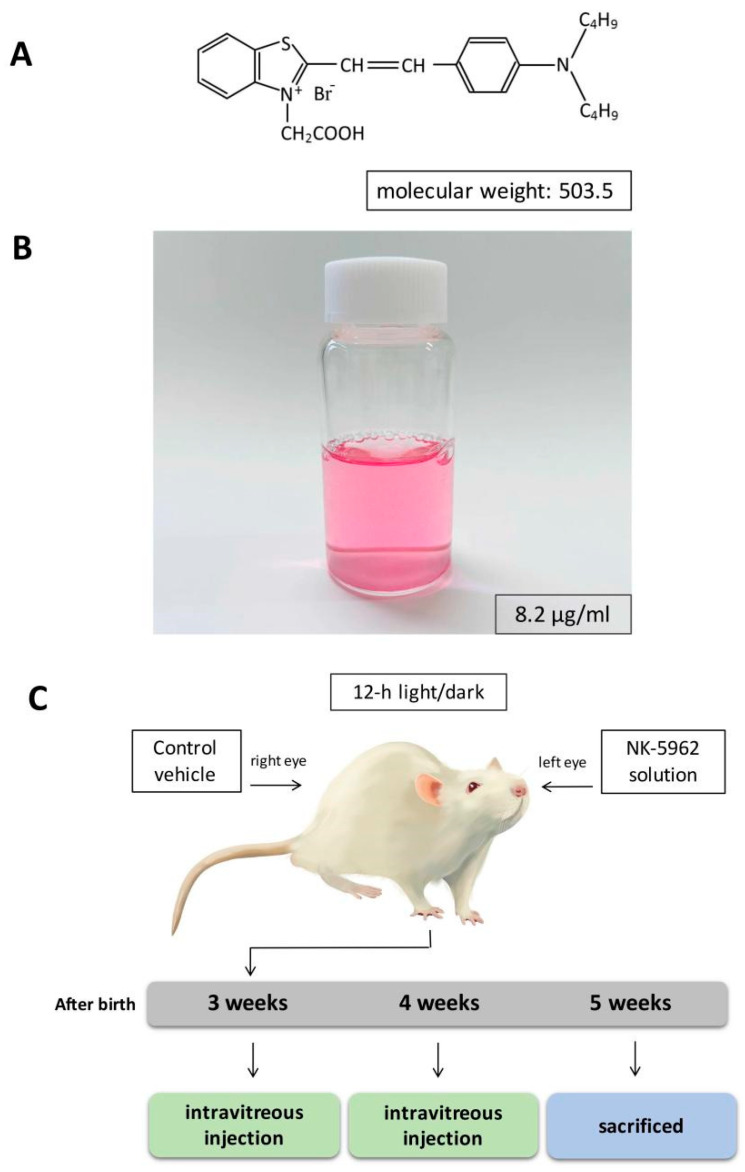
NK-5962 and experimental design. (**A**) Chemical structure of NK-5962. (**B**) NK-5962 solution (8.2 μg/mL). (**C**) Experimental schedule.

The photoelectric dye NK-5962 (Figure 1A), 2-[2-[4-(dibutylamino)phenyl]ethenyl]-3-carboxymethylbenzothiazolium bromide, generates electric potential in response to light [18,19]. We previously developed an Okayama University-type retinal prosthesis (OUReP™), which is composed of NK-5962-coupled polyethylene thin films, and showed that OUReP™ evokes neuronal response by light stimulation [20,21]. We found that the NK-5962 molecule itself protected both neural retinal cells and RPE cells from apoptosis through the primary mixed culture of retinal cells, NK-5962 coupled film transplanted into the eyes of RCS rats, and intravitreal injection of NK-5962 solution in RCS rats [22,23,24]. We recently demonstrated that NK-5962 shows low levels of reactive oxygen species (ROS) generation and that its phototoxicity is very low. These findings suggest that NK-5962 is a good candidate for the treatment of RP [25].

In this study, we aimed to explore the mechanisms involved in the anti-apoptotic effect of intravitreal injection of NK-5962 in RCS rats by RNA-seq and bioinformatics analyses [26].

## 2. Results

### 2.1. Screening of DEGs in the Eyes Injected with NK-5962

**Figure 2:**
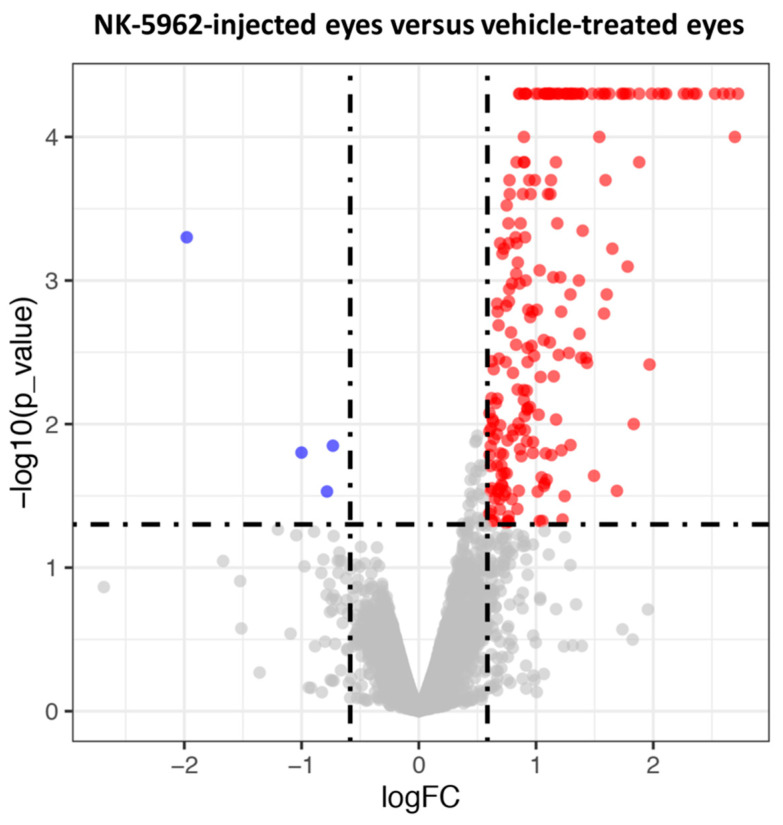
The volcano plot shows the distribution of the fold changes of each mRNA transcript in NK-5962-injected eyes versus vehicle-treated eyes. Genes that pass a threshold of log(FC)  >  0.585, *p* value < 0.05 are highlighted by red (up-regulated) and blue (down-regulated), respectively. Only one gene (*LYVE1*) was commonly down-regulated among samples treated with NK-5962. FC: fold change.

In order to reveal the mechanism of NK-5962 in attenuating retinal cell apoptosis, we examined the changes in gene expression between NK-5962-treated and control groups by RNA-seq analysis. The total number of reads per sample ranged from 46.2 million to 68.6 million. We only focused on the genes with FPKM (fragments per kilobase million) >0.1 in each group to avoid genes with low expression. Genes with log(FC) ≥ 0.672 and a *p* value < 0.05 were selected for follow-up studies. Volcano plots show the global transcriptional changes in NK-5962-injected eyes versus vehicle-treated eyes at the age of 5 weeks (Figure 2). Totally, 55 genes (Table 1) were chosen as up-regulated DEGs in the eyes treated with NK-5962. According to the *p* values and log(FC) values, Serpin Family F member 1 (*SERPINF1*) was found to be the most significantly up-regulated gene in NK-5962-treated retinas compared with the controls (Table 1). By contrast, we found a gene—the *LYVE1* gene—that was commonly down-regulated among samples treated with NK-5962 (Table 2).

### 2.2. Bioinformatics Analysis of DEGs in the Eyes Injected with NK-5962

**Figure 3:**
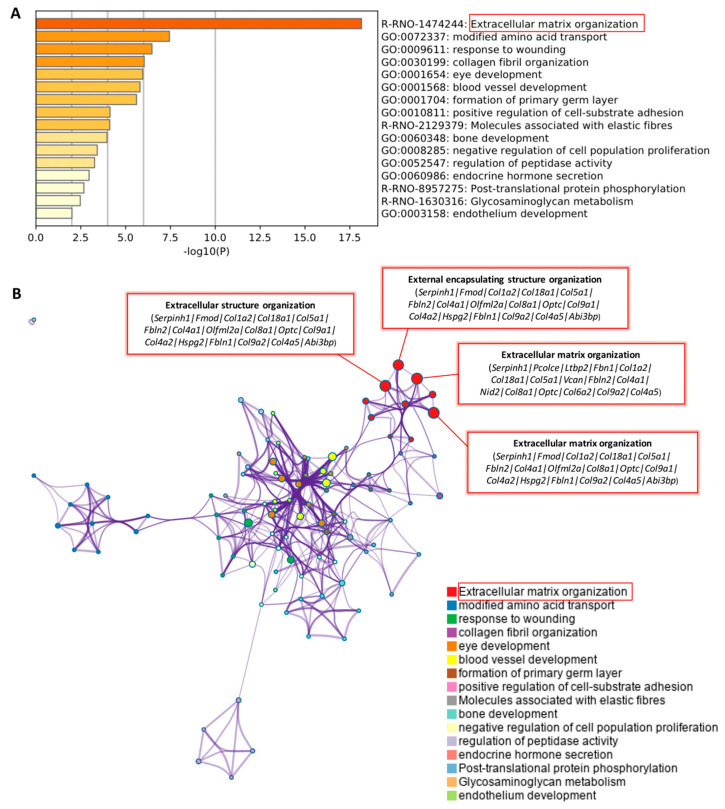
The enrichment analysis of 55 significant up-regulated genes was performed by Metascape. (**A**) Metascape bar graph for viewing the top enriched clusters, where each cluster uses a discrete color to indicate statistical significance. (**B**) Metascape visualization of the interactome network formed by all 55 genes from the Table 1, where the MCODE compounds are colored according to their identities. The most interesting enriched terms in the category were extracellular matrix organization (red box).

The functional annotation and pathway enrichment analysis of 55 up-regulated DEGs (Table 1) were explored by using GO terms, KEGG pathway, and Reactome pathway analyses in the Metascape database (Figure 3A,B). All GO terms and pathways can be seen online (See Supplementary Table S1 online). Then, we checked the relevant literature to find GO terms and pathways related to anti-apoptosis in the eyes treated with NK-5962. As shown in Figure 3A, enrichment analysis by Metascape showed that most of the DEGs were significantly enriched in the extracellular matrix organization pathway (red box and Table 3).

In addition, the network was visualized by Cytoscape, where each node means an enriched term. A red box shows extracellular matrix-related pathways and genes, such as extracellular matrix organization, extracellular structure organization, and external encapsulating structure organization (Figure 3B, Table 4).

The results of the KEGG pathway analysis (Metascape) showed that the up-regulated DEGs were significantly enriched in the ECM-receptor interaction and *PI3K–Akt* signaling pathway (Table 5).

**Figure 4:**
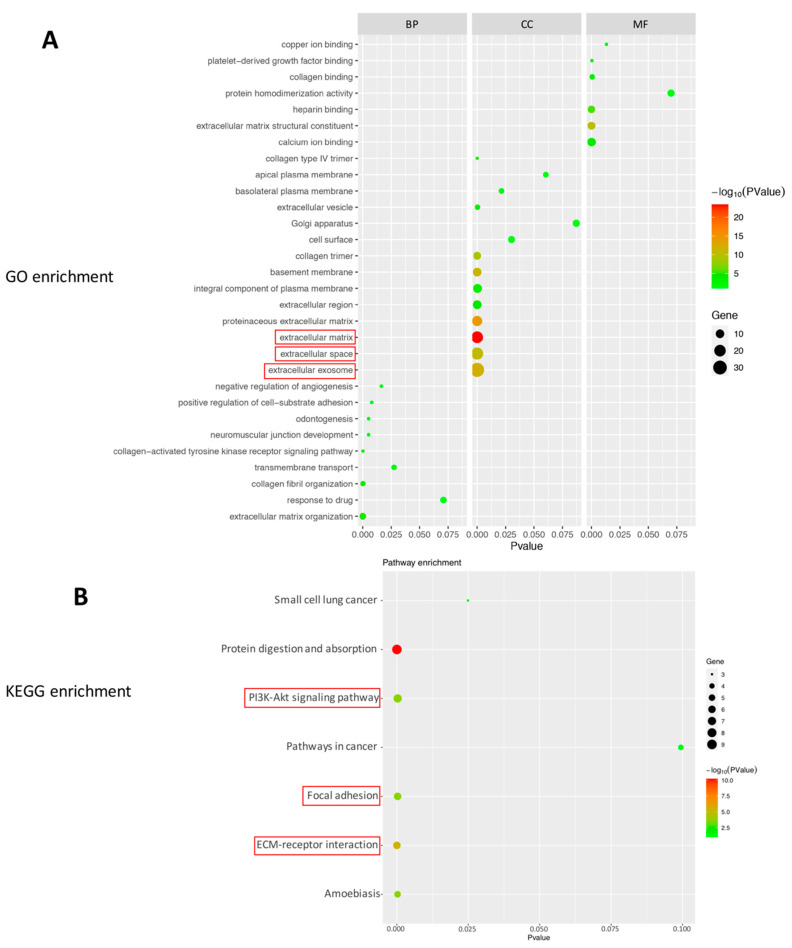
Enrichment analysis of top 55 up-regulated genes based on DAVID bioinformation resources. (**A**) Bubble plot of the enriched GO terms: cellular component terms (CC), molecular function terms (MF), biological process terms (BP). The first three pathways with the most genes (smaller *p* value), and which may be related to protection of photoreceptor cells are as follows: extracellular exosome, extracellular space, and extracellular matrix (red box). (**B**) Bubble plot of the enriched KEGG pathways. The pathways which may be related to protection of photoreceptor cells are as follows: *PI3K–Akt* signaling pathway. In addition, there are *PI3K–Akt* signaling pathway-related pathways: focal adhesion, ECM-receptor interaction (according to the map of *PI3K–Akt* signaling pathway, https://www.genome.jp/kegg-bin/show_pathway?rno04151 (accessed on 18 June 2021). The colors of the nodes are illustrated from red to green in descending order of –log10 (*p* value). X-axis: signaling pathway or function; Y-axis: percentage of the number of DEGs assigned to a term among the total number of DEGs annotated in the network; Bubble size: number of DEGs assigned to a pathway or function; Color: enriched *p* value.

We also uploaded the 55 DEGs (Table 1) into DAVID bioinformation resources for functional annotation analysis. Based on smaller *p* values and greater number of genes contained therein, the up-regulated genes indicated that the proteins of biological process (BP) were associated with extracellular matrix organization. With regard to the cellular component (CC), the majority of proteins contained extracellular exosome (including 31 genes, *p* value = 8.19 × 10^−13^), extracellular space (including 22 genes, *p* value = 2.30 × 10^−11^), and extracellular matrix (including 21 genes, *p* value = 3.45 × 10^−24^). With regard to molecular function (MF), the majority of proteins were involved in processes such as, extracellular matrix structural constituent (Figure 4A, Table 6).

Additionally, the up-regulated 55 genes were enriched in five KEGG pathways (DAVID), including the *PI3K–Akt* signaling pathway, ECM-receptor interaction, focal adhesion, protein digestion and absorption, and amoebiasis (Figure 4B, Table 7). The first three pathways are related to anti-apoptosis mechanisms.

## 3. Discussion

This study aimed to investigate the mechanisms of photoelectric dye NK-5962 in delaying the apoptosis of retinal neurons. We used RCS rats as a retinitis pigmentosa model, which show progressive photoreceptor degeneration as the consequence of *MERTK* mutation in the RPE cells [86]. Our results show that NK-5962 produces an effect on the expression of a variety of genes. These include genes involved in regulating the *PI3K–Akt* signaling pathway and inhibiting the apoptosis of photoreceptor cells in RCS rats.

First, we found that both Metascape and DAVID analyses showed a lot of extracellular matrix (ECM)-related terms in NK-5962-injected eyes. The ECM of the retina is divided into two separate entities: the interphotoreceptor matrix (IPM) and the retinal ECM. During retinal degeneration, the ECM structure is destroyed, leading to an acceleration of the retinal degeneration process. These changes would lead to an increase in the space between the cells and a reduction in the ECM materials that were required to support the retina. In turn, it would change the delivery of oxygen, growth factors, and nutrients from the retinal supply to the photoreceptor cells [87]. The effectiveness of drug treatment would be based on healthy retinal ECM so that neurotrophic factors may play the role in protecting photoreceptor cells [88]. We speculate that NK-5962 maybe postpone retinal cell degeneration by up-regulating ECM-related pathways to support the RPE-photoreceptor microenvironment and to provide an optimal microenvironment for viability of neurons.

Second, the extracellular exosome term that contained the highest number of genes in GO analysis using DAVID in this study was one of the subtypes of extracellular vesicles (EVs). EVs can reach injured and degenerative neural cells quickly and transfer biologically active substances directly into cells [89,90]. The recent research found that inhibited synthesis of extracellular exosomes leads to exacerbation of retinal degeneration. In mice that are depleted of extracellular exosomes, inflammation and cell death increases, and retinal function decreases after photo-oxidative damage occurs [91]. We speculate that the anti-apoptotic effect of NK-5962 in the retina of RCS may be mediated by extracellular exosomes, which release neurotrophic factors, lipids, and proteins, including PEDF and *SOD3*, promoting the survival of photoreceptors and maintaining the homeostasis of the retinal microenvironment.

Furthermore, in our study, the *PI3K–Akt* signaling pathway, focal adhesion pathway, and ECM-related pathways were up-regulated by NK-5962 in the KEGG pathway analysis using DAVID. According to the KEGG pathway map of *PI3K–Akt* signaling pathway–Norway rats (Rattus norvegicus), NK-5962 maybe activate *PI3K–Akt* signaling pathway through focal adhesion and ECM-receptor interaction pathway. Previous reports showed that *PI3K–Akt* pathway protected the survival of cone photoreceptors [92]. Additionally, we noticed that the genes involved in the *PI3K–Akt* signaling pathway were collagen genes and the *FGFR2* gene (Table 7). The *FGFR2* gene is a factor that mediates the rescue of photoreceptors in the rat and has an effect on anti-apoptotic and neurite repair [93,94]. These results indicate that the delivery of NK-5962 maybe protect photoreceptors from apoptosis in RCS rat through up-regulated *FGFR2* gene by activating the *PI3K–Akt* signaling pathway. All of these possibilities need to be clarified through further research.

On the basis of *p* values and fold change values, the first gene to be noticed is *SERPINF1*, which encodes PEDF. PEDF is a multifunctional protein that has neurotrophic [95] and antioxidant properties [96] as well as an anti-inflammatory role [97]. PEDF is also known to protect photoreceptors from injury in rd10 mouse models of retinal degeneration [27,98]. The other reviews showed that molecular pathways of retinal survival activity triggered by PEDF are involved in *PI3K–Akt* [99]. The other gene we focused on is *SOD3*, which was up-regulated after injection of NK-5962. In recent studies, it has been shown that *SOD3* is important in protecting the ECM from oxidative damage [100]. Whether the translation of these genes has also been changed remains to be verified.

This study showed the potential mechanism of NK-5962, with a protective effect at the early stage of photoreceptor degeneration in RCS rats by RNA-seq. In the next step, to locate the position of up-regulated genes in NK-5962-treated eyes, we will perform RT-PCR and multicolor immunostaining experiments to screen out important genes.

## 4. Methods

### 4.1. Chemicals and Preparations

NK-5962 was obtained from Hayashibara, Inc. (Okayama City, Japan) (Figure 1A), and was dissolved in distilled deionized water at a concentration of 8.2 μg/mL (16 μM) (Figure 1B).

### 4.2. Animals

All experiments were performed in compliance with the ARVO statement for the “Use of Animals in Ophthalmology and Vision Research” and were approved by the Animal Care and Use Committee at Okayama University (Identifier OKU-2019196). Eight male pink-eyed RCS (Jcl-rdy/rdy, p-) rats were obtained from CLEA Japan, Inc. (Tokyo, Japan), and reared under a 12 h light/dark cycle. All intravitreal injections were performed as described previously [6]. At the age of 3 and 4 weeks, the rats were anesthetized by intraperitoneal injection of ketamine (87 mg/kg body weight, Daiichi Sankyo, Tokyo, Japan) and xylasine (13 mg/kg, Bayer Japan, Osaka, Japan), and received an intravitreal injection of 5 μL of NK-5962 solution at 8.2 μg/mL (16 μM) in the left eye, and saline (0.9% sodium chloride) as a vehicle control in the right eye, with a 30-gauge needle-attached Hamilton syringe (50 μL 1705 LT SYR; Hamilton Company, Reno, NV, USA) under a dissecting microscope. All rats were sacrificed at the age of 5 weeks (Figure 1C).

### 4.3. RNA Extraction

Neural retinal tissue was dissected free from the other tissues of the eye and stored in an RNAlater RNA Stabilization Reagent (Cat# 74104, Qiagen, Germany). Total RNA was extracted from the dissected retinal tissue using an RNeasy Mini Kit (Cat# 74104, Qiagen, Germany) combined with a QIAshredder kit and RNase-free DNase Set (Qiagen) as per the manufacturer’s instructions.

### 4.4. RNA Sequencing

Total RNA samples were submitted to Macrogen Japan (Tokyo) and Riken Genesis (Tokyo) for bioanalyzer quality control analysis (QC), Illumina next-generation sequencing (NGS), and differential expressed gene (DEG) analysis. All submitted samples had an RNA integrity number (RIN) > 9 and were proceeded for library construction. The sequencing library was prepared from poly-A selected RNA from each sample with TruSeq Stranded mRNA Library Prep Kit (Illumina). On the platform of Novaseq 6000 System (Illumina) and HiSeq 2500 (Illumina), transcriptome sequencing was performed (100 bp paired-end sequencing). Adaptor sequences and low-quality bases from paired-reads were removed by Cutadapt (version 2.4). Filtered paired end reads were mapped to the rat reference genome (UCSU rn4) by HISAT2 (version 2.1.0), and then transcript assembly was performed by Cufflinks (v2.1.1) using a previously defined rat gene annotation [101]. Cuffdiff in the Cufflinks package was used to identify DEGs. RNA-seq was performed on three independent sample sets, and genes that showed reproducible changes in three experiments were used for bioinformatics analysis. The *p* values were calculated by combining the reads of the three experiments. A cutoff fold-change (FC) ≥ 1.3 and *p* value < 0.05 were assumed to identify genes significantly changed by NK-5962 treatment.

### 4.5. Bioinformatics Analysis

Identified DEGs were uploaded to Metascape (https://metascape.org/, accessed on 8 June 2021), which facilitates comparative analyses of multiple datasets, gene ontology (GO) annotation, Kyoto Encyclopedia of Genes and Genomes (KEGG), and Reactome pathway enrichment analyses. The database for Annotation, Visualization, and Integrated Discovery (DAVID, v6.8) bioinformatics tool (https://david.ncifcrf.gov, accessed on 8 June 2021) was also used for validating the results. GO and KEGG bioinformatic analyses were conducted in R 3.6.3 (https://cran.r-project.org/ (accessed on 28 June 2021). Volcano plots were created using the R-package ggplot2 (https://cran.r-project.org/ (accessed on 28 June 2021).

### 4.6. Data Availability

The datasets presented in this study can be found in online repositories. The raw data obtained in this study are available from DDBJ Read Archive (https://ddbj.nig.ac.jp//DRASearch/ (accessed on 9 December 2021) under accession numbers of (DRA013172) for RNA-seq.

## 5. Conclusions

We found that NK-5962 up-regulated several genes involved in extracellular matrix organization, extracellular exosome, and *PI3K–Akt* signaling pathways in RCS rats. Additionally, we observed the up-regulation of PEDF, which has been reported to prevent photoreceptor cells death. In order to further elucidate the molecular mechanisms of the anti-apoptotic properties of NK-5962 in a rat model of RP, more in-depth research is needed. These are very important for the development of new therapeutic agents for patients with retinal degenerative diseases.

## Figures and Tables

**Table 1:** Up-regulated genes in NK-5962-treated retinas.

Gene Name	Description	Locus	Log2(Fold_Change)	*p*_Value	*q*_Value	References
*SERPINF1*	Serpin Family F Member 1	chr10:62713440-62739444	2.722	5.00 × 10^−5^	0.012	[27,28]
*COL4A1*	Collagen Type IV Alpha 1 Chain	chr16:83045182-83157835	2.651	5.00 × 10^−5^	0.012	[29]
*CRYAB*	Crystallin Alpha B	chr8:54107289-54111502	2.368	5.00 × 10^−5^	0.012	[30]
*COL4A2*	Collagen Type IV Alpha 2 Chain	chr16:82899293-83045155	2.293	5.00 × 10^−5^	0.012	[31]
*HSPG2*	Heparan Sulfate Proteoglycan 2	chr5:156226988-156328912	2.089	5.00 × 10^−5^	0.012	[32]
*AQP1*	Aquaporin 1	chr4:84098345-84110524	2.043	5.00 × 10^−5^	0.012	[33]
*ANXA1*	Annexin A1	chr1:223478435-223494455	1.798	5.00 × 10^−5^	0.012	[34]
*Ecrg4*	ECRG4 augurin precursor	chr9:42930953-42950605	1.575	5.00 × 10^−5^	0.012	[35]
*WLS*	Wnt Ligand Secretion Mediator	chr2:258014377-258128180	1.392	5.00 × 10^−5^	0.012	[36]
*SLC22A8*	Solute Carrier Family 22 Member 8	chr1:211269365-211287596	1.388	5.00 × 10^−5^	0.012	[37]
*SOD3*	Superoxide dismutase 3	chr14:63381446-63387180	1.328	5.00 × 10^−5^	0.012	[38,39]
*FBLN2*	Fibulin 2	chr4:125380499-125441075	1.296	5.00 × 10^−5^	0.012	[40]
*OPTC*	Opticin	chr13:46846755-46858100	1.292	5.00 × 10^−5^	0.012	[41]
*SLC13A4*	Solute Carrier Family 13 Member 4	chr4:62679592-62724547	1.265	5.00 × 10^−5^	0.012	[42]
*FGFR2*	Fibroblast Growth Factor Receptor 2	chr1:189482974-189589279	1.243	5.00 × 10^−5^	0.012	[43]
*FBLN1*	Fibulin 1	chr7:123208153-123287289	1.194	5.00 × 10^−5^	0.012	[44]
*TYRP1*	Tyrosinase-Related Protein 1	chr5:99518305-99537289	1.190	5.00 × 10^−5^	0.012	[45]
*OGN*	Osteoglycin	chr17:20969065-21145330	1.160	5.00 × 10^−5^	0.012	[46]
*GJA1*	Gap Junction Protein Alpha 1	chr20:35409814-35422259	1.117	5.00 × 10^−5^	0.012	[47]
*WFDC1*	WAP Four-Disulfide Core Domain 1	chr19:49924309-49943113	1.116	5.00 × 10^−5^	0.012	[48]
*LTBP2*	Latent Transforming Growth Factor Beta Binding Protein 2	chr6:108826438-108924895	1.112	5.00 × 10^−5^	0.012	[49]
*COL4A5*	Collagen Type IV Alpha 5 Chain	chrX:36918650-37130562	1.105	5.00 × 10^−5^	0.012	[50]
*DAPL1*	Death-Associated Protein Like 1	chr3:41187966-41207910	1.070	5.00 × 10^−5^	0.012	[51]
*ENPP2*	Ectonucleotide Pyrophosphatase/Phosphodiesterase 2	chr7:91295814-91377947	0.997	5.00 × 10^−5^	0.012	[52]
*SLC13A3*	Solute Carrier Family 13 Member 3	chr3:156447899-156510620	0.914	5.00 × 10^−5^	0.012	[53]
*MXRA8*	Matrix Remodeling Associated 8	chr5:172698112-172702607	0.899	5.00 × 10^−5^	0.012	[54]
*COL9A1*	Collagen Type IX Alpha 1 Chain	chr9:22907067-22990836	0.855	5.00 × 10^−5^	0.012	[55]
*COL8A1*	Collagen Type VIII Alpha 1 Chain	chr11:43604973-43737050	1.879	1.50 × 10^−4^	0.029	[56]
*MFRP*	Membrane Frizzled-Related Protein	chr8:47084055-47089218	1.169	1.50 × 10^−4^	0.029	[57]
*COL5A1*	Collagen Type V Alpha 1 Chain	chr3:6825780-6973521	0.901	1.50 × 10^−4^	0.029	[58]
*FBN1*	Fibrillin 1	chr3:112607811-112804951	0.895	1.50 × 10^−4^	0.029	[59]
*COL18A1*	Collagen alpha-1(XVIII) chain	chr20:11872458-11982466	0.834	1.50 × 10^−4^	0.029	[60]
*SLC6A13*	Solute Carrier Family 6 Member 13	chr4:157736263-157771945	0.942	2.00 × 10^−4^	0.036	[61]
*ABI3BP*	ABI Family Member 3 Binding Protein	chr11:44853363-45072422	1.122	2.50 × 10^−4^	0.041	[62]
*CPXM1*	Carboxypeptidase X, M14 Family Member 1	chr3:118000979-118007777	1.102	2.50 × 10^−4^	0.041	[63]
*FMOD*	Fibromodulin	chr13:46987713-46998331	0.887	2.50 × 10^−4^	0.041	[64]
*VCAN*	Versican	chr2:19712628-19812592	0.868	4.00 × 10^−4^	0.061	[44]
*SERPINH1*	Serpin Family H Member 1	chr1:156666873-156674336	0.765	4.00 × 10^−4^	0.061	[65]
*PCOLCE*	Procollagen C-Endopeptidase Enhancer	chr12:19672504-19690374	1.398	4.50 × 10^−4^	0.068	[66]
*SLC26A4*	Solute Carrier Family 26 Member	chr6:49389211-49427000	0.835	5.50 × 10^−4^	0.078	[67]
*FSTL1*	Follistatin Like 1	chr11:64680819-64735683	0.694	5.50 × 10^−4^	0.078	[68]
*OLFML2A*	Olfactomedin Like 2A	chr3:18731164-18751940	0.713	6.50 × 10^−4^	0.089	[69]
*MRC2*	Mannose Receptor C Type 2	chr10:94689060-94753073	0.831	9.00 × 10^−4^	0.117	[70]
*GSTM2*	Glutathione S-Transferase Mu 2	chr2:203549021-203553380	1.207	9.50 × 10^−4^	0.120	[71,72]
*COL6A2*	Collagen Type VI Alpha 2 Chain	chr20:12436782-12464512	0.859	1.05 × 10^−3^	0.127	[73]
*COL9A2*	Collagen Type IX Alpha 2 Chain	chr5:141623364-141640224	0.770	1.15 × 10^−3^	0.137	[74]
*NID2*	nidogen-2	chr15:4801182-4856895	0.769	1.40 × 10^−3^	0.163	[75,76]
*F5*	Coagulation Factor V	chr13:79934955-79997282	0.745	1.50 × 10^−3^	0.171	[77]
*SNED1*	Sushi, Nidogen, and EGF-Like Domains 1	chr9:92509498-92568597	0.672	1.65 × 10^−3^	0.181	[78]
*COLEC12*	Collectin Subfamily Member 12	chr18:996296-1188288	0.951	1.80 × 10^−3^	0.192	[79]
*COL1A2*	Collagen Type I Alpha 2 Chain	chr4:29393502-29429101	1.066	2.60 × 10^−3^	0.264	[80]
*SLC16A12*	Solute Carrier Family 16 Member 12	chr1:238643039-238665699	0.962	2.85 × 10^−3^	0.281	[81]
*CLDN19*	Claudin 19	chr5:139838013-139842711	0.896	5.80 × 10^−3^	0.480	[82]
*MYO5C*	Myosin VC	chr8:80042255-80118773	0.921	5.85 × 10^−3^	0.481	[83]
*PMEL*	Premelanosome Protein	chr7:2007881-2045336	1.294	1.40 × 10^−2^	0.941	[84]

**Table 2:** Down-regulated genes in NK-5962-treated retinas.

Gene Name	Description	Locus	Log2(Fold_Change)	*p*_Value	*q*_Value	Reference
*LYVE1*	Lymphatic Vessel Endothelial Hyaluronan Receptor 1	chr1:168601459-168622234	−1.001	1.58 × 10^−2^	0.999	[85]

**Table 3:** Top Reactome pathways significantly enriched in DEGs related to anti-apoptosis in NK-5962-treated retinas (Metascape).

Category	Term	Description	LogP	InTerm_ InList	Genes
Reactome Gene Sets	R-RNO- 1474244	Extracellular matrix organization	−18.264	16/198	*Serpinh1*, *Pcolce*, *Ltbp2*, *Fbn1*, *Col1a2*, *Col18a1*, *Col5a1*, *Vcan*, *Fbln2*, *Col4a1*, *Nid2*, *Col8a1*, *Optc*, *Col6a2*, *Col9a2*, *Col4a5*, *Fmod*, *Olfml2a*, *Col9a1*, *Col4a2*, *Hspg2*, *Fbln1*, *Abi3bp*, *Fgfr2*

**Table 4:** Top enriched GO terms significantly enriched in DEGs related to anti-apoptosis in NK-5962-treated retinas (Metascape).

Category	Term	Description	LogP	InTerm_ InList	Genes
GO Biological Processes	GO:0030198	extracellular matrix organization	−16.615	17/308	*Serpinh1*, *Fmod*, *Col1a2*, *Col18a1*, *Col5a1*, *Fbln2*, *Col4a1*, *Olfml2a*, *Col8a1*, *Optc*, *Col9a1*, *Col4a2*, *Hspg2*, *Fbln1*, *Col9a2*, *Col4a5*, *Abi3bp*
GO Biological Processes	GO:0043062	extracellular structure organization	−16.591	17/309	*Serpinh1*, *Fmod*, *Col1a2*, *Col18a1*, *Col5a1*, *Fbln2*, *Col4a1*, *Olfml2a*, *Col8a1*, *Optc*, *Col9a1*, *Col4a2*, *Hspg2*, *Fbln1*, *Col9a2*, *Col4a5*, *Abi3bp*
GO Biological Processes	GO:0045229	external encapsulating structure organization	−16.567	17/310	*Serpinh1*, *Fmod*, *Col1a2*, *Col18a1*, *Col5a1*, *Fbln2*, *Col4a1*, *Olfml2a*, *Col8a1*, *Optc*, *Col9a1*, *Col4a2*, *Hspg2*, *Fbln1*, *Col9a2*, *Col4a5*, *Abi3bp*

**Table 5:** Top KEGG pathways significantly enriched in DEGs related to anti-apoptosis in NK-5962-treated retinas (Metascape).

Category	Term	Description	LogP	InTerm_InList	Genes
KEGG Pathway	ko04512, rno04512	ECM-receptor interaction	−9.901	8/81	*Col1a2*, *Col4a1*, *Col9a1*, *Col4a2*, *Hspg2*, *Col6a2*, *Col9a2*, *Col4a5*
KEGG Pathway	ko04151, rno04151	*PI3K–Akt* signaling pathway	−5.166	8/329	*Fgfr2*, *Col1a2*, *Col4a1*, *Col9a1*, *Col4a2*, *Col6a2*, *Col9a2*, *Col4a5*

**Table 6:** Top three GO terms significantly enriched in DEGs related to anti-apoptosis in NK-5962-treated retinas (DAVID).

Category	Term	Count	%	*p* Value	Genes
GOTERM_ CC_DIRECT	GO:0070062~ extracellular exosome	31	56.3	8.19 × 10^−13^	*COLEC12*, *COL18A1*, *SNED1*, *LTBP2*, *FBLN1*, *FBLN2*, *FSTL1*, *NID2*, *AQP1*, *GJA1*, *SERPINH1*, *SLC13A3*, *GSTM2*, *ANXA1*, *SERPINF1*, *SLC6A13*, *PCOLCE*, *SOD3*, *HSPG2*, *COL1A2*, *COL4A2*, *COL5A1*, *COL6A2*, *OGN*, *MYO5C*, *MXRA8*, *COL8A1*, *SLC26A4*, *SLC22A8*, *CRYAB*, *FBN1*
GOTERM_ CC_DIRECT	GO:0005615~ extracellular space	22	40.0	2.30 × 10^−11^	*COL18A1*, *ANXA1*, *SERPINF1*, *RGD1305645*, *WFDC1*, *PCOLCE*, *LTBP2*, *FBLN1*, *SOD3*, *FSTL1*, *HSPG2*, *F5*, *VCAN*, *COL1A2*, *ABI3BP*, *COL6A2*, *OGN*, *SERPINH1*, *ENPP2*, *CPXM1*, *FMOD*, *FBN1*
GOTERM_ CC_DIRECT	GO:0031012~ extracellular matrix	21	38.1	3.45 × 10^−24^	*COL18A1*, *SERPINF1*, *PCOLCE*, *LTBP2*, *FBLN1*, *SOD3*, *NID2*, *HSPG2*, *FBLN2*, *VCAN*, *COL1A2*, *COL4A2*, *COL5A1*, *COL4A1*, *ABI3BP*, *COL6A2*, *OGN*, *COL8A1*, *FMOD*, *FGFR2*, *FBN1*

**Table 7:** Top three KEGG pathways significantly enriched in DEGs related to anti-apoptosis in NK-5962-treated retinas (DAVID).

Category	Term	Count	%	*p* Value	Genes
KEGG_PATHWAY	rno04151:*PI3K–Akt* signaling pathway	7	12.7	2.76 × 10^−4^	*COL1A2*, *COL4A2*, *COL5A1*, *COL4A1*, *COL6A2*, *COL4A5*, *FGFR2*
KEGG_PATHWAY	rno04512:ECM- receptor interaction	6	10.9	4.03 × 10^−6^	*COL1A2*, *COL4A2*, *COL5A1*, *COL4A1*, *COL6A2*, *COL4A5*
KEGG_PATHWAY	rno04510:Focal adhesion	6	10.9	2.52 × 10^−4^	*COL1A2*, *COL4A2*, *COL5A1*, *COL4A1*, *COL6A2*, *COL4A5*

## Data Availability

The datasets presented in this study can be found in online repositories. The raw data obtained in this study are available from DDBJ Read Archive (https://ddbj.nig.ac.jp//DRASearch/, accessed on 9 December 2021) under the accession number (DRA013172) for RNA-seq.

## Supplementary Materials

The following are available online at https://www.mdpi.com/article/10.3390/ijms222413276/s1.
